# Predicting intestinal viability by consecutive photoacoustic monitoring of oxygenation recovery after reperfusion in acute mesenteric ischemia in rats

**DOI:** 10.1038/s41598-021-98904-x

**Published:** 2021-09-30

**Authors:** Takumi Sugiura, Kenichiro Okumura, Junichi Matsumoto, Maki Sakaguchi, Takahiro Komori, Takahiro Ogi, Dai Inoue, Wataru Koda, Satoshi Kobayashi, Toshifumi Gabata

**Affiliations:** 1 Department of Radiology, Kanazawa University Graduate School of Medical Sciences, 13-1 Takara-machi, Kanazawa, Ishikawa 920-8641 Japan; 2 Department of Diagnostic Pathology, Kanazawa University, 13-1 Takara-machi, Kanazawa, Ishikawa 920-8641 Japan; 3 Department of Quantum Medical Technology, Kanazawa University Graduate School of Medical Sciences, 5-11-80 Kodatsuno, Kanazawa, Ishikawa 920-0942 Japan

**Keywords:** Gastroenterology, Medical research

## Abstract

The purpose was to assess whether consecutive monitoring of oxygenation by photoacoustic imaging (PAI) can objectively predict intestinal viability during surgery for acute mesenteric ischemia (AMI). PAI uses laser light to detect relative amounts of oxygenated and deoxygenated hemoglobin in intestinal tissue. In 30 rats, AMI was induced by clamping the mesenteric and marginal vessels of the ileum for 0 min in the control group, 30 min in the mild group, and 180 min in the severe group (10 rats per group). After 60 min of reperfusion, intestinal damage was evaluated pathologically. Oxygenation of the intestine was monitored throughout the procedure in real time by a commercially available PAI system and compared among the groups. All rats showed irreversible (*i.e.* transmucosal or transmural infarction) damage in the severe group. After reperfusion, the oxygenation in the mild group recovered immediately and was significantly higher than in the severe group at 1, 5, 10, 30, and 60 min (*P* = .011, 002, < .001, 001, and 001, respectively). Oxygenation showed a significant strong negative correlation with pathological severity (r_s_ =  − 0.7783, − 0.7806, − 0.7422, − 0.7728, and − 0.7704, respectively). In conclusion, PAI could objectively predict irreversible ischemic damage immediately after reperfusion, which potentially prevents inadequate surgery.

## Introduction

During surgery for acute mesenteric ischemia (AMI), since there is no reliable objective marker of intestinal viability to determine the resection margin, it is assessed subjectively by the surgeon. However, this requires a high level of clinical experience^1–4^. Because of that, second-look surgery without closing the abdomen for about 2 days is an alternative approach, but this is quite invasive to the patient^5^.

In recent years, photoacoustic imaging (PAI), also known as optoacoustic imaging, has been demonstrating its usefulness in a variety of fields, including clinical practice^6–14^. It is an emerging real-time in vivo imaging modality that combines optical imaging contrast with ultrasonic spatial resolution. PAI uses a wide range of the spectrum including the near-infrared spectrum, which has deep penetration and visualizes deep tissue in vivo in real time. Light energy is selectively absorbed by endogenous and exogenous chromophores in tissue causing rapid thermoelastic expansion, which generates broadband acoustic waves that can be detected by ultrasound transducers^15^. Differing absorption spectra of oxygenated hemoglobin and deoxyhemoglobin with dual-wavelength PAI allow quantification of the blood oxygen level. The usefulness of oxygenation mapping in organ ischemia has also been reported^16–18^.

There are several reports of the usefulness of PAI in assessing intestinal viability^19–21^. Recently, Wang et al. reported the usefulness of PAI for detecting early intestinal ischemia–reperfusion injury in rats^20^. They reported that, in a model of superior mesenteric artery embolism, there was a significant increase of PAI signal intensity in the group with a certain degree of damage compared to the control group. However, more objective and quantitative markers to predict intestinal viability are desired. To the best of our knowledge, the usefulness of monitoring oxygenation after reperfusion in the evaluation of intestinal viability has not yet been reported.

With PAI, oxygenation of ischemic intestine after reperfusion can be evaluated noninvasively during surgery. In this study, a homogeneous intestinal ischemia model of strangulated bowel obstruction, which is more frequently encountered clinically, was created in rats. We hypothesized that real-time consecutive monitoring of oxygenation after reperfusion could contribute to the determination of the resection margin by predicting intestinal viability. The purpose of this study was to evaluate the usefulness of oxygenation mapping by PAI to objectively and quantitatively predict intestinal viability during surgery for AMI.

## Results

### Laboratory tests

**Figure 1:**
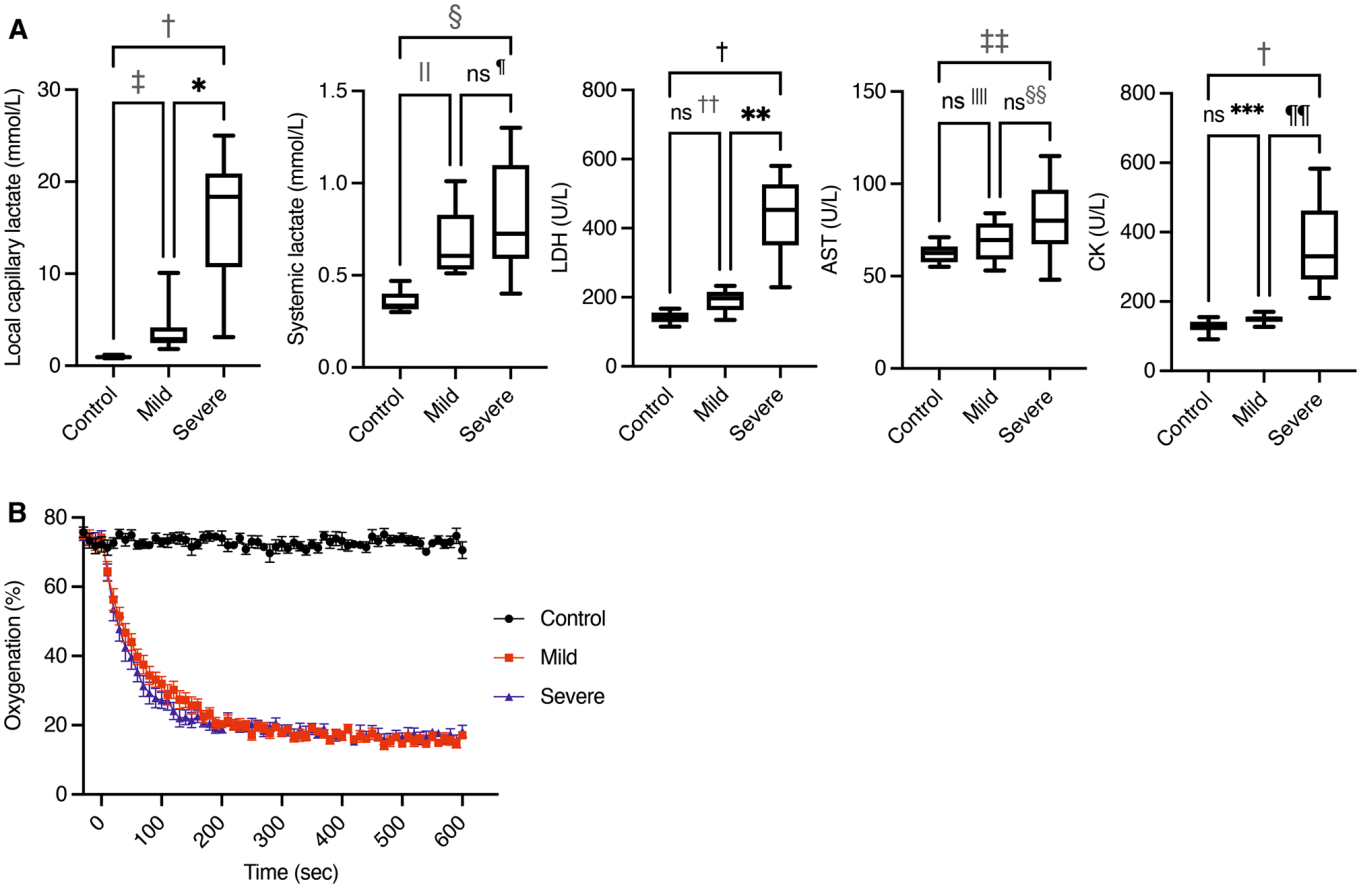
(**A**) Results of laboratory tests in the three groups (n = 10 rats per group). Note: Values are expressed as box and whiskers plots; the minimum and the maximum value (whiskers), and the 25th and 75th percentile (box), and median. Control, control group; Mild, mild acute mesenteric ischemia group; Severe, severe acute mesenteric ischemia group; LDH, lactate dehydrogenase; AST, aspartate transaminase; CK, creatine phosphokinase; ns, not significant; ^✻^*P* = .0283; ^✝^*P* < .0001; ^ǂ^*P* = .0437; ^§^*P* = .0001; ^❘^^❘^*P* = .0023; ^¶^*P* > .9999; ^✻^^✻^*P* = .0204; ^✝^^✝^*P* = .1083; ^ǂ^^ǂ^*P* = .0237; ^§^^§^*P* = .4318; ^❘^^❘^^❘^^❘^*P* = .6969; ^¶^^¶^*P* = .0104; ^✻^^✻^^✻^*P* = .2260. The Kruskal–Wallis test was used for evaluation. (**B**) Time course of mean oxygenation of the control, mild, and severe groups in the early stage of AMI. The mean oxygenation of the three groups from 30 s before clamping to 10 min after clamping. A rapid decrease of oxygenation from baseline (> 70%) after clamping is clearly visualized. The oxygenation reaches a plateau within 5 min. Oxygenation of the mild and severe group does not show a significant difference at baseline (0 min), 1, 5, and 10 min after clamping. Note: Error bars represent standard errors of the mean (n = 10 rats per group).

Results of laboratory tests are shown in Fig. 1A. Local capillary lactate was significantly higher in the severe group (18.35 (3.10–25.00) mmol/L) than in the mild group and the control group (2.90 (1.80–10.10) and 0.95 (0.80–1.20) mmol/L, *P* = 0.0283 and < 0.0001, respectively). There was also a significant difference between the mild group and the control group (*P* = 0.0437). Systemic lactate was significantly higher in the severe group and in the mild group (0.73 (0.40–1.30) and 0.61 (0.51–1.01) mmol/L) than in the control group (0.34 (0.30–0.47) mmol/L, *P* = 0.0001 and 0.0023, respectively), but there was no significant difference between the severe group and the mild group (*P* > 0.9999). Lactate dehydrogenase (LDH) (severe, 453.0 (229.0–580.0); mild, 197.0 (134.0–233.0); control, 139.0 (115.0–167.0) U/L) was significantly higher in the severe group than in the others (severe vs control, *P* < 0.0001; severe vs mild, *P* = 0.0204), but there was no significant difference between the mild and control groups (*P* = 0.1083). Creatine phosphokinase (CK) (severe, 330.5 (210.0–583.0); mild, 151.0 (127.0–170.0); control, 125.5 (91.0–155.0) U/L) was also significantly higher in the severe group than in the others (severe vs control, *P* < 0.0001; severe vs mild, *P* = 0.0104), but there was no significant difference between the mild and control groups (*P* = 0.2260). Aspartate transaminase (AST) was significantly higher in the severe group (80.0, (48.0–115.0) U/L) than in the control group (62.5 (55.0–71.0) U/L, *P* = 0.0237), but there was no significant difference between the mild group (69.5 (53.0–84.0) U/L) and the severe group or the control group (mild vs severe, *P* = 0.4318; mild vs control, *P* = 0.6969).

### Time course of oxygenation in AMI

**Figure 2:**
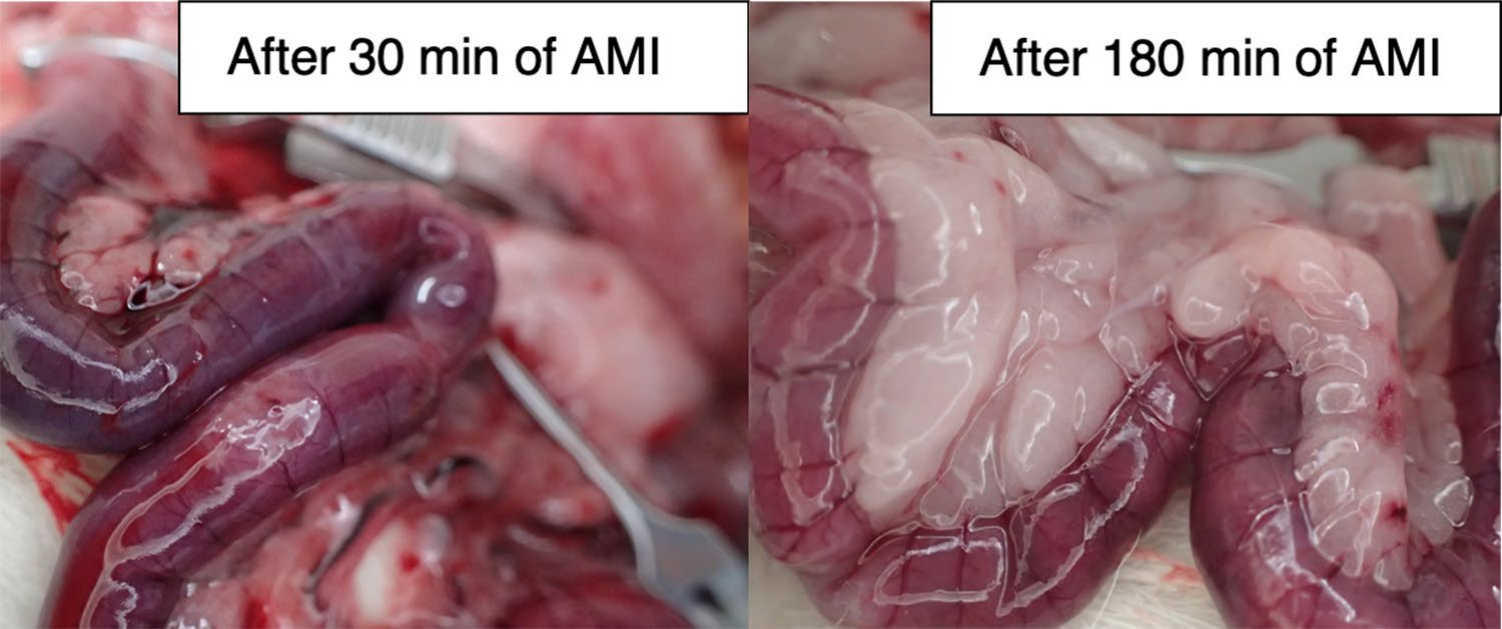
Gross appearance of ischemic ileum in the mild and severe groups. In some cases, the severity of AMI is indistinguishable by the gross appearance alone. PAI is evaluated in the middle of the ischemic region by attaching the transducer on the proximal wall, and five adjacent regions of interest are set in the cross-sectional image (for example, see Fig. 5B). Note: AMI, acute mesenteric ischemia; PAI, photoacoustic imaging.

Before clamping, average oxygenation was greater than 70% in the three groups. All rats in the mild and severe groups showed a rapid decrease of oxygenation after clamping and then reached a plateau (< 30%) within five minutes. The time course of the oxygenation before and after clamping is shown in Fig. 1B. Before clamping, there was no significant difference among the three groups (control, 70.5 (63.0–82.8); mild, 72.3 (68.4–79.4); severe, 76.4 (64.7–80.0); all *P* > 0.9999). After clamping, oxygenation of the mild and severe groups was significantly lower than that of the control group (mild vs control, 38.0 (27.5–50.3) vs 71.4 (66.8–78.1); *P* = 0.0017 at 1 min, 17.4 (12.7–24.4) vs 72.7 (61.5–82.7); *P* = 0.0002 at 5 min and 16.8 (13.0–24.0) vs 70.8 (53.0–81.1); *P* = 0.0003 at 10 min after clamping; severe 34.4 (22.6–50.3) vs control 71.4 (66.8–78.1); *P* < 0.0001 at 1 min, 19.5 (11.7–26.4) vs 72.7 (61.5–82.7); *P* = 0.0008 at 5 min and 17.2 (10.8–28.1) vs 70.8 (53.0–81.1); *P* = 0.0006 at 10 min after clamping). There were no significant differences between the mild and severe groups at 1, 5, and 10 min after clamping (all *P* > 0.9999). The appearance of ischemic intestine was dark, and it tended to be darker in the severe group, but it was not always distinguishable between the two ischemic groups (Fig. 2).

**Figure 3:**
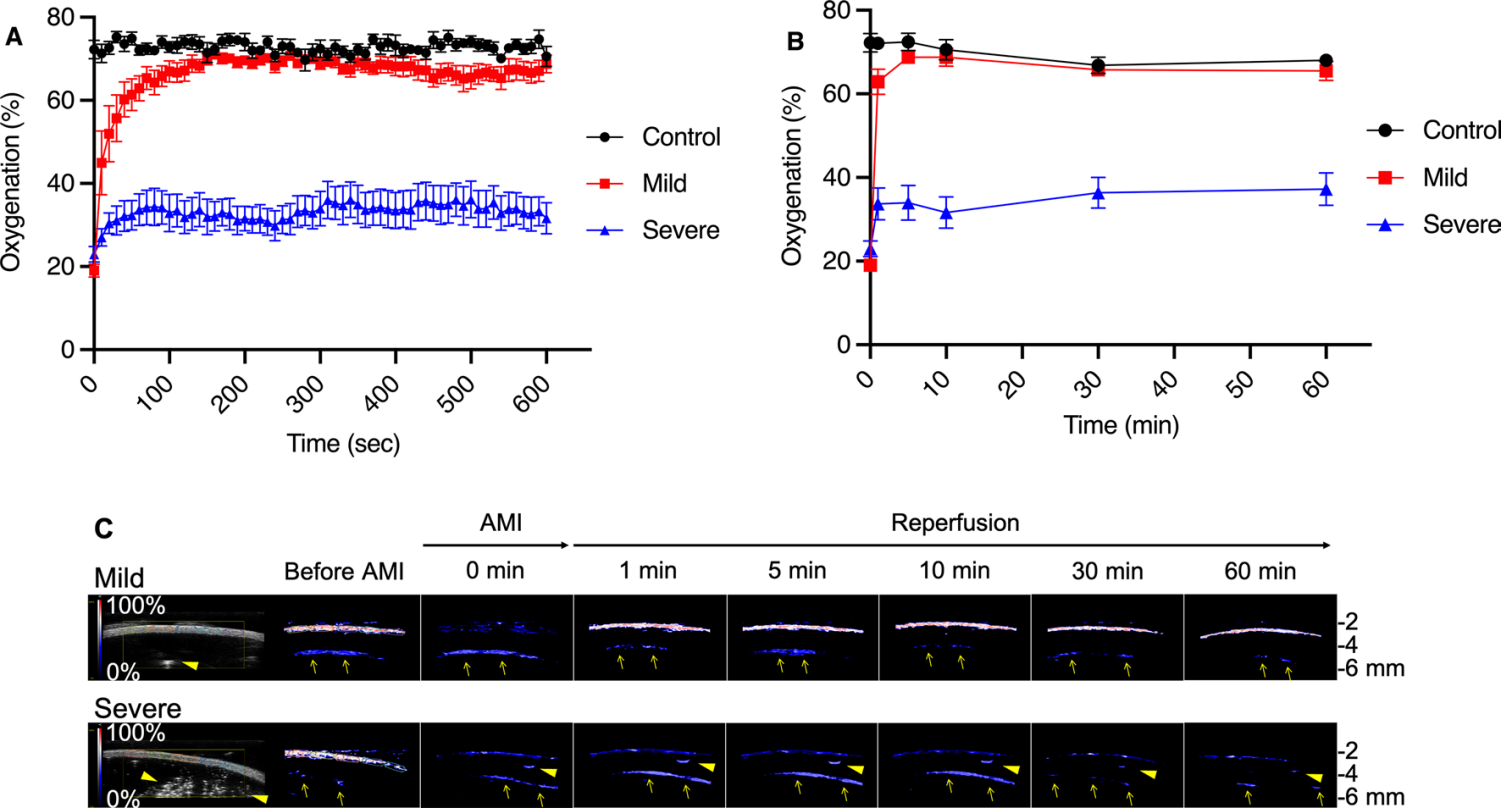

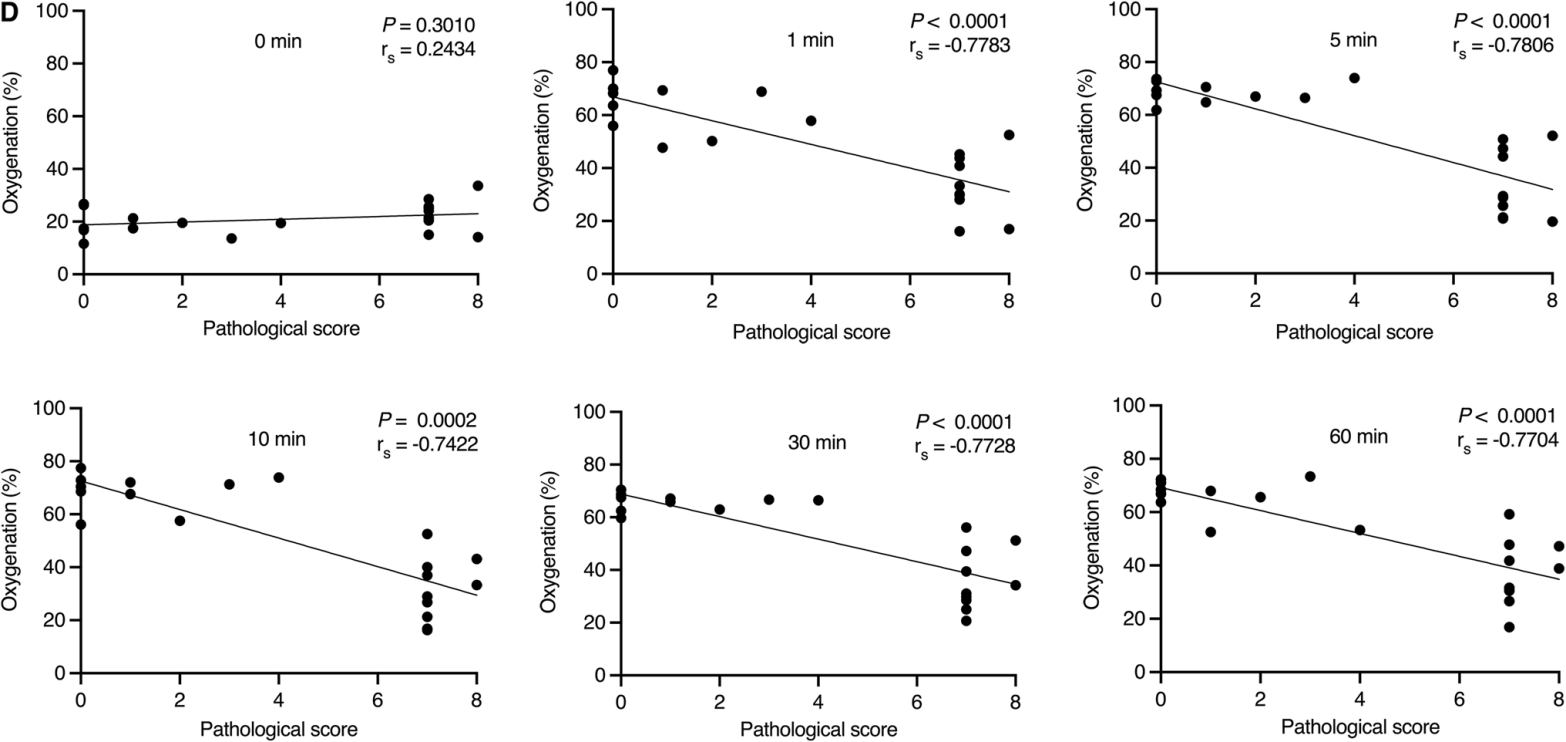
(**A**) Time course of mean oxygenation of the three groups until 10 min after reperfusion. The mild AMI group shows rapid recovery of oxygenation to the baseline within one minute, whereas the severe AMI group shows insufficient recovery. Note: Error bars represent standard errors of the mean (n = 10 rats per group). (**B**) Time course of mean oxygenation of the three groups until 60 min after reperfusion. Oxygenation of the mild group was significantly higher than that of the severe group at 1, 5, 10, 30, and 60 min after reperfusion. Note: Error bars represent standard errors of the mean (n = 10 rats per group). (**C**) Photoacoustic imaging of the proximal wall of the distal ileum before inducing AMI, before and 1, 5, 10, 30, and 60 min after reperfusion by removing vascular clamps in the mild and severe groups. B-mode image and oxygenation mapping of the mild and severe groups are shown on the left side. The oxygenation ranges from 0% (dark blue) to 100% (red). In both groups, oxygenation mapping shows equally high oxygenation at baseline (before AMI), and equally low oxygenation after a predetermined duration of AMI (30 min in the mild group and 180 min in the severe group). In the mild group, oxygenation clearly recovers 1 min after reperfusion. On the other hand, in the severe group, oxygenation does not apparently recover 1 min after reperfusion, and does not recover even after 60 min. Five adjacent regions of interest are placed on the proximal wall (shown on B-mode images). Arrows show reverberation artifact from the proximal wall, and arrowheads show reverberation artifact from air bubbles within ultrasound gel on the bowel surface. Note: AMI, acute mesenteric ischemia; Mild, the mild group; Severe, the severe group; Before AMI, baseline; 0 min, after a predetermined duration of AMI (30 min in the mild group, 180 min in the severe group) and just before reperfusion (removing vascular clamps); 1 min, 1 min after reperfusion (removing vascular clamps); 5 min, 5 min after reperfusion; 10 min, 10 min after reperfusion; 30 min, 30 min after reperfusion; 60 min, 60 min after reperfusion; numbers shown on the right side indicate the depth. (**D**) Linear regression of oxygenation at 0, 1, 5, 10, 30, and 60 min after reperfusion against the pathological score of the mild and severe groups (total 20 rats). At all time points after reperfusion, there is a strong negative linear relationship between oxygenation and the pathological score. The correlation coefficient at 1 min after reperfusion is already comparable to those at other time points. The correlation coefficient (r_s_) and *P*-values are shown on the upper right corner of each figure. Spearman’s test was used for evaluation.

Figure 3A,B, and Table 1 show the time course of oxygenation in the three groups after reperfusion. Before removing the vascular clamps, there was no significant difference between the mild and severe groups. Once the vascular clamps were removed, oxygenation recovered to the baseline level (about 65–75%) immediately in the mild group. On the other hand, oxygenation recovered slightly but remained lower in the severe group (Fig. 3C). At 1, 5, 10, 30, and 60 min after the removal of vascular clamps, oxygenation was significantly lower in the severe group than in the mild group (Table 1).

**Table 1:** Oxygenation after reperfusion in the control, mild, and severe groups.

Time (min)	Oxygenation in the control group	Oxygenation in the mild group	Oxygenation in the severe group	*P*-value		
				Control vs Mild	Control vs Severe	Mild vs Severe
0	70.5 (63.0–82.8)	18.4 (11.6–26.7)	22.9 (14.1–33.7)	< .0001	.0029	.9287
1	71.4 (66.8–78.1)	65.9 (47.7–77.0)	31.7 (16.1–52.6)	.3121	< .0001	.0105
5	72.7 (61.5–82.7)	68.4 (61.9–73.9)	29.0 (19.6–52.2)	> .9999	< .0001	.0022
10	70.8 (53.0–81.1)	70.9 (56.1–77.4)	31.1 (16.3–52.5)	> .9999	.0002	.0007
30	67.4 (51.3–74.7)	66.6 (59.7–70.5)	32.6 (20.7–56.1)	> .9999	.0002	.0014
60	68.0 (62.7–75.8)	67.4 (52.5–73.3)	35.3 (16.9–59.2)	> .9999	.0003	.0011

Note: Oxygenation of the mild and severe groups before and after removing the vascular clamps and the control group are shown. The time course of oxygenation after 1 min of reperfusion was clearly different between the mild and severe groups. Oxygenation was significantly higher in the mild group than in the severe group at 1, 5, 10, 30, and 60 min and did not show a significant difference with the control group. All values are shown as median (min–max). The Kruskal–Wallis test was used for evaluation.

### Pathological findings

In the severe group, all tissue samples showed irreversible damage, and the pathological score was 7 (7–8), significantly higher than in the other groups (control vs severe, *P* < 0.0001, mild vs severe, *P* = 0.0018). In the mild group, the pathological score was 0.5 (0–4). In the control group, the pathological score was 0 in all rats, and there was no significant difference between it and the mild group (*P* = 0.5098). In the severe group, dilated capillaries, thrombi and congestion were observed in the small veins, which caused congestion.

### Correlation analysis between oxygenation and the pathological score

Correlation analysis between oxygenation and the pathological score was performed (Fig. 3D). Figure 3D shows Spearman’s correlation coefficient (r_s_-value) along with the corresponding *P*-values for the correlation between oxygenation and the pathological score at each time point, showing a significant strong negative correlation at each time point after reperfusion. The r_s_-value at 1 min after reperfusion was already comparable to those at the other time points.

## Discussion

Recently, the usefulness of PAI as an objective and quantitative evaluation method after ischemia and reperfusion has been widely reported in various organs including intestine^20,22,23^, and the feasibility of evaluating oxygenation by PAI has been established^24^.

During AMI surgery, intestinal viability is subjectively assessed by surgeons and is highly dependent on their experience. Karliczek et al. evaluated surgeons’ predictive accuracy for anastomotic leakage in gastrointestinal surgery, but the predictive value was low in a prospective, clinical study^25^. Incorrect evaluation may result in bowel stricture, obstruction, necrosis, anastomotic leakage, and short bowel syndrome^2,25^.

In the present study, there was a significant difference in the time course of oxygenation immediately after reperfusion between the two ischemic groups with different prognoses. At the same time, it is important to note that, just before reperfusion (after a predetermined duration of AMI), there was no significant difference in oxygenation between the two groups. In addition, oxygenation in the intestine at each time point showed a significant strong negative correlation between the pathological scores, even at 1 min after reperfusion. To the best of our knowledge, this is the first report to evaluate intestinal viability in AMI with consecutive monitoring of oxygenation after reperfusion by PAI. These results indicate that consecutive monitoring of oxygenation by PAI, which, similar to ultrasound, can be performed at the bedside, may serve as an objective and quantitative marker to determine the resection margin immediately after reperfusion during surgery for AMI. LDH, CK and local capillary lactate suggested the severe damage, but their usefulness is limited because they cannot be measured in the segment of interest or are highly dependent on the amount of ischemic bowel.

### Oximetry with Photoacoustic imaging

Singh et al. showed that the serosal oxygenation measurements were not sensitive to ischemia^26^. It has been estimated that blood flow in the mucosal and submucosal layers accounts for 70% of total blood flow in intestinal tissues; therefore, microcirculation should be evaluated in the mucosal and submucosal layers^27^. There are various methods to evaluate intestinal viability during surgery, and most of them reflect oxygenation and perfusion from the serosal surface, but none of them has become a clinical standard due to various deficiencies^2,28^. As mentioned above, PAI can be performed during surgery. In humans, intestinal oxygenation can be measured intraoperatively using PAI with its high spatial resolution without any contrast media^29^. With this method, oxygenation of the layers including the mucosal and submucosal layers can be evaluated noninvasively. In principle, oxygenation evaluation can be performed within tens of milliseconds, in other words, motion artifacts are almost negligible unlike computed tomography or magnetic resonance imaging^20,30^. Intraoperative guidance by PAI is greatly awaited in various fields^31^.

### Real-time consecutive monitoring of oxygenation

In the present study, before inducing AMI, the average oxygenation of normal ileum was between 70 and 80%, which was compatible with the previous report using visual light spectroscopy^25^. In the early stage of AMI, oxygenation reached a plateau within five minutes after inducing AMI (Fig. 1B). From the present result, intestinal viability cannot be predicted by the absolute oxygenation value itself before reperfusion. This finding is clinically acceptable. Decreased staining of the intestinal wall on contrast-enhanced computed tomography, which cannot be performed in patients with allergies or renal dysfunction, is a key finding for diagnosing AMI, but an intestinal segment with decreased staining is not always irreversibly damaged after reperfusion. At the same time, we have demonstrated the ability of PAI to detect severe intestinal ischemia at 1 min after reperfusion compared to the mild group without contrast media. In clinical practice, second-look surgery for AMI after reperfusion without closing the abdomen to clarify the intestinal viability and determine the precise resection margin is an option, however, that is quite invasive to the patient^5^. The results of this study may reduce the invasiveness.

The decrease in oxygenation can be caused either by a decrease in arterial blood with rich oxygenation or by possible pooling of venous blood. The significantly low oxygenation after reperfusion in the severe AMI group may due to vasospasm, thrombosis, and congestion in damaged intestinal tissue^32^. In the present study, dilated capillaries, thrombi, and congestion were observed in the severe group, which caused congestion. It is also known that hypoxia is induced in inflamed tissues by increased metabolic demand and oxygen consumption^33–35^.

The present study has some limitations. First, in strangulated bowel obstruction in clinical practice, venous flow is compromised prior to arterial flow. In the present model, both arterial and venous flow were obstructed simultaneously. Second, the reason why oxygenation decreased was not specifically clarified, although the disruption of the oxygenated blood supply and the congestion of the intestinal wall are thought to be the cause of hypoxia. Also, increased metabolic demand and oxygen consumption are thought to be the cause. Moreover, there are histological differences in the intestine between rats and humans; thus, the result cannot be immediately applied translationally^36^.

## Conclusion

In the present study, real-time consecutive monitoring of oxygenation after reperfusion using PAI showed that the oxygenation of the reversibly damaged group recovered quickly to baseline levels, whereas the oxygenation of the irreversibly damaged group showed insufficient recovery. There was a significant difference in oxygenation recovery between the two groups at only 1 min after reperfusion and the oxygenation after reperfusion showed a strong negative correlation with the pathological damage score. These results indicate that consecutive monitoring of oxygenation may make irreversible ischemic tissue damage predictable during surgery for AMI, and it can help determine the resection margin objectively and quantitatively, and potentially prevent inadequate surgery and invasive management.

## Methods

### Animal models

Thirty male Sprague–Dawley rats provided by Charles River Laboratories Japan (Yokohama, Japan) were studied. All animal experiments were performed in accordance with the guidelines of our institution and approved by our animal research committee (study protocol ID: AP194060). Rats were maintained in a specific pathogen-free facility. All rats weighed approximately 600 g and had ad libitum access to food and water. AMI was established by the procedure described below.

### Rat model of AMI

**Figure 4:**
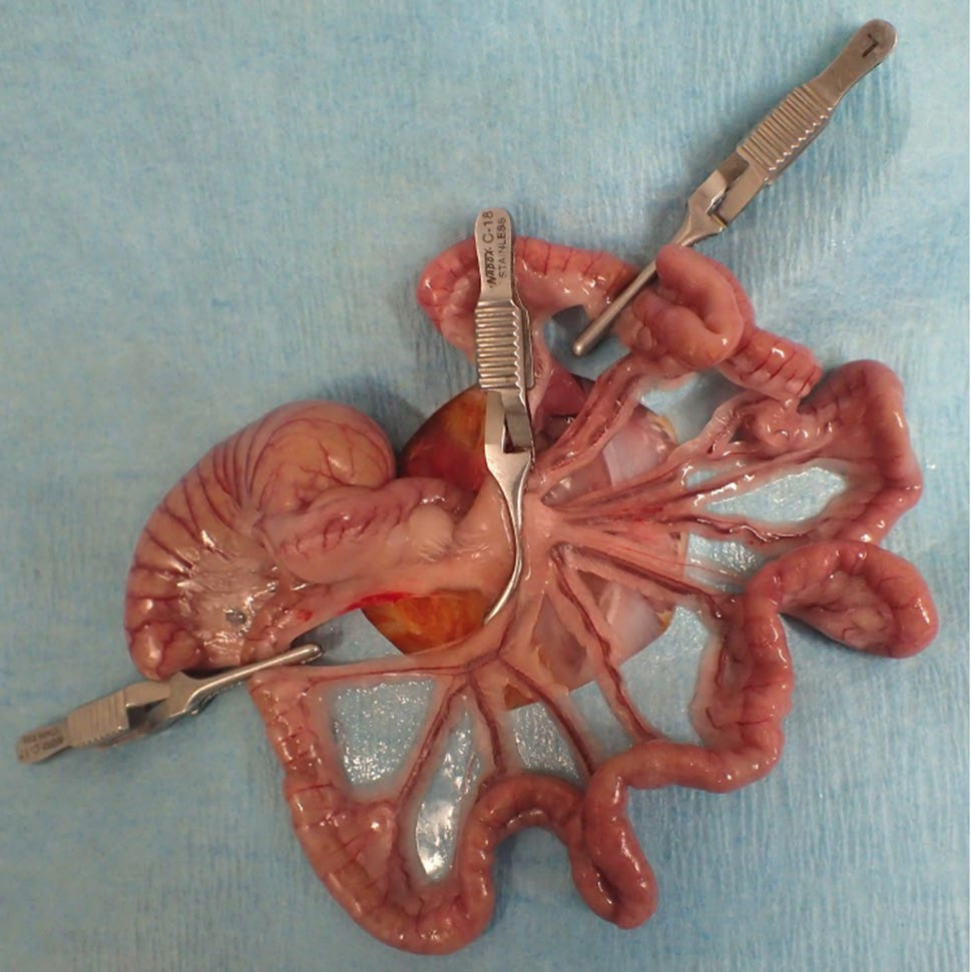
Strangulated bowel obstruction model in rats. Acute mesenteric ischemia is induced in 15 cm of ileum from the ileocecal valve using vascular clamps. Mesenteric arteries and veins and marginal arteries and veins are clamped.

**Figure 5:**
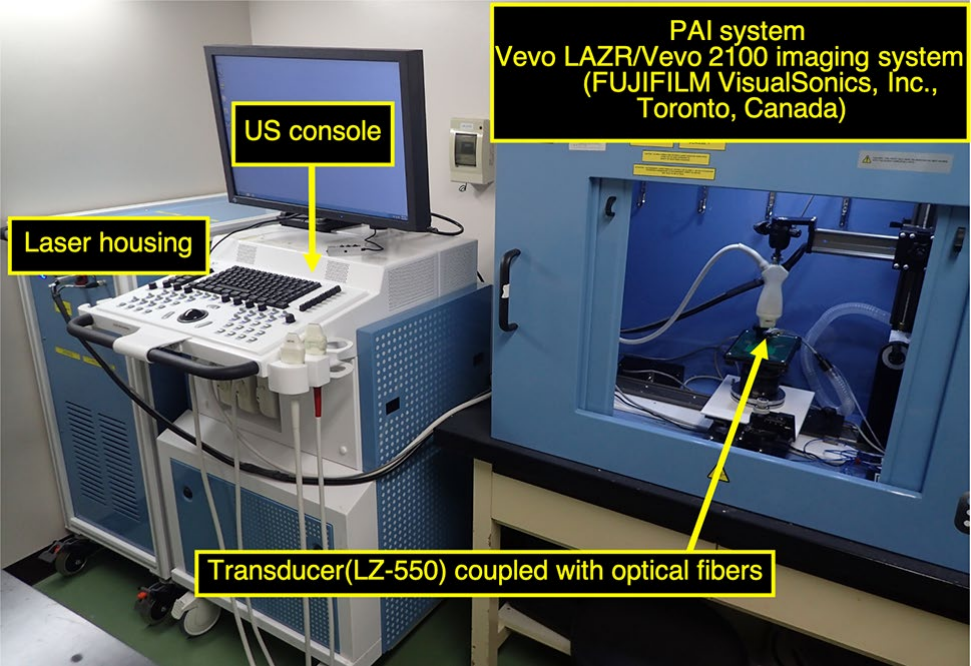
Commercially available photoacoustic imaging systems. Vevo LAZR uses handheld transducers for laser delivery and photoacoustic signal detection.

After 24 h of fasting, the abdomen was shaved. A 3-cm midline incision was made, and a 15-cm length of ileum from the ileocecal valve was exposed. Blood samples were obtained from the femoral vein to evaluate systemic lactate, LDH, AST, and CK levels. Subsequently, local capillary lactate was evaluated by puncturing the intestine. The rats were randomly assigned to three groups in advance. The first group was the control group (n = 10), which was designed to assess the effect of anesthesia and the invasiveness of the operation to standardize the study in terms of ischemia. The second group was the mild AMI group (n = 10), in which mesenteric arteries and veins and marginal arteries and veins were clamped with vascular clamps, and mesenteric ischemia was then induced in 15 cm of ileum from the ileocecal valve (strangulated bowel obstruction model; Fig. 4); after 30 min of ischemia, the clamps were removed, and blood samples were obtained, and local capillary lactate was again evaluated; after 60 min of reperfusion, a tissue sample was obtained for pathological evaluation. The third group was the severe AMI group (n = 10), which was the same as the mild AMI group, but with 180 min of ischemia. During AMI and reperfusion, oxygenation of ischemic intestine was monitored by a commercially available PAI system, Vevo LAZR/Vevo 2100 (FUJIFILM VisualSonics Inc., Toronto, Canada, Fig. 5.

Induction of anesthesia was performed with 5% isoflurane, and it was then maintained by intraperitoneal injection of a mixture of medetomidine hydrochloride (0.15 mg/kg), midazolam (2.0 mg/kg), and butorphanol tartrate (2.5 mg/kg). Vital signs (heart rate, respiratory rate, peripheral oxygen saturation) were continuously monitored by MouseOx PLUS (Starr Life Sciences Co., Oakmont, PA, USA) to exclude hypoventilation. The neck of each rat was also shaved, and a clip sensor was affixed. During the procedure, the rats were kept on a heating pad to avoid hypothermia.

### Ultrasound and PAI

PAI was performed using a Vevo LAZR/2100 imaging system with an LZ-550 linear-array transducer (256 elements, center frequency 40 MHz, bandwidth 27 MHz) used to acquire all images. The tunable laser supplied 10–20 mJ per pulse over the 680–970 nm wavelength range, with a pulse repetition frequency of 10 Hz. Once initialized, the system was switched to “Oxy-Hemo” mode to obtain parametric maps of oxygenation using the following parameters: depth 6 mm; width 14.08 mm; and wavelengths 750 and 850 nm. Oxygenation mapping was shown with B-mode images (Fig. 3C). The transducer was attached to the proximal intestinal wall via a sterile ultrasound gel. To reduce artifacts generated by the intestinal contents, the ultrasound gel was also injected into the lumen of the intestine. When AMI was induced, oxygenation of the three groups was compared before clamping and 1, 5, and 10 min after clamping. Oxygenation was measured as the average of five adjacent regions of interest placed on the cross-sectional image of the proximal wall for 1 s (Fig. 3C). After a predetermined duration of AMI (30 min in the mild group and 180 min in the severe group), oxygenation of the three groups was compared with the values before the removal of vascular clamps, and 1, 5, 10, 30, and 60 min after removal.

### Pathological findings

**Figure 6:**
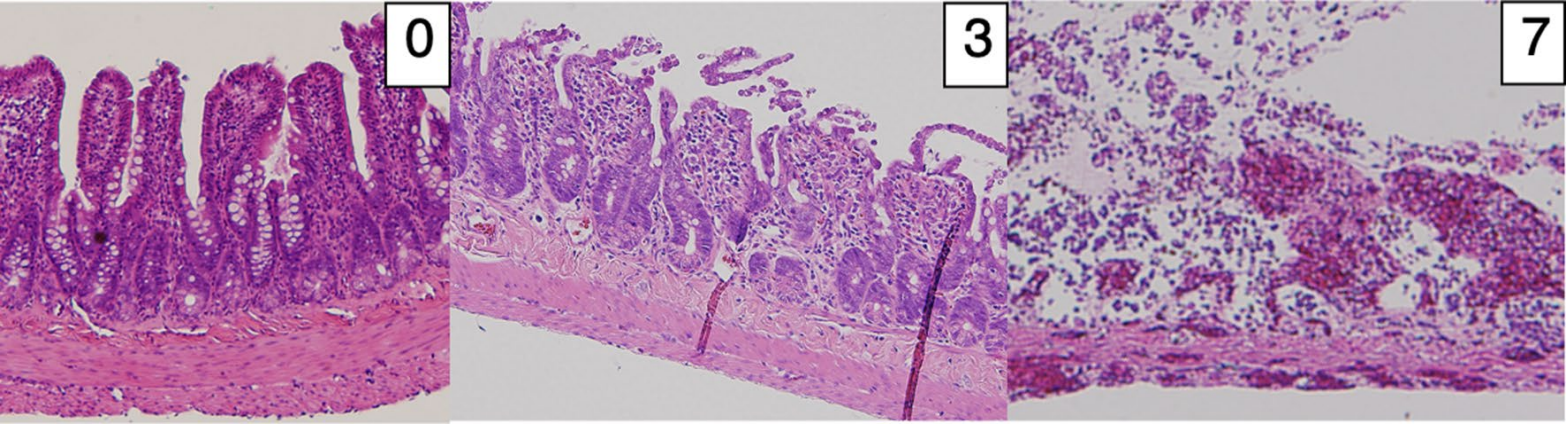
Examples of pathological findings with the pathological scores. 0, normal mucosa; 3, massive lifting down sides of villi, some denuded tips; 7, transmucosal infarction. With score 7, thrombi in small veins are seen.

At the end of each experiment, a tissue sample was obtained for histopathological study. Tissues were stained with hematoxylin and eosin and evaluated by a pathologist blinded to group assignment. The sections from the intestine were classified according to the degree of tissue injury in accordance with Chiu’s score classification including modifications proposed by Park^37,38^. This Park/Chiu’s classification of small intestinal injury consists of values from 0 to 8, which are shown in Table 2. Samples of pathological findings are shown in Fig. 6. Scores of 7 and 8 were considered to indicate irreversible ischemic damage. The correlation between the pathological score and oxygenation at each time point after reperfusion was evaluated.

**Table 2:** Scoring system for assessment of ischemic damage.

Grade	Description
0	Normal mucosa
1	Subepithelial space at villus tips
2	Extension of subepithelial space with moderate lifting
3	Massive lifting down sides of villi, some denuded tips
4	Denuded villi, dilated capillaries
5	Disintegration of lamina propria
6	Crypt layer injury
7	Transmucosal infarction
8	Transmural infarction

Note: A commonly used pathological classification system for ischemic mucosal lesions proposed by Chiu et al.^37^, including modifications proposed by Park et al.^38^.

### Statistical analysis

All results are expressed as median (min–max) unless otherwise noted. Statistical analysis and graphical displays of data were performed using GraphPad software (version 9.1.1 for Mac; GraphPad Software, San Diego, CA, USA). The Kruskal–Wallis test and Dunn’s multiple comparisons test were performed to evaluate the values of laboratory tests and oxygenation among the groups. Correlation was evaluated by linear regression and Spearman’s correlation coefficient (r_s_-value). A *P*-value < 0.05 was considered significant.

## Abbreviations

AMI: Acute mesenteric ischemia
PAI: Photoacoustic imaging
LDH: Lactate dehydrogenase
CK: Creatine phosphokinase
AST: Aspartate transaminase
